# Organotypic Cultures as a Model to Study Adult Neurogenesis in CNS Disorders

**DOI:** 10.1155/2016/3540568

**Published:** 2016-04-05

**Authors:** Fabio Cavaliere, Monica Benito-Muñoz, Carlos Matute

**Affiliations:** Departamento de Neurociencias, Achucarro Basque Center for Neuroscience, Universidad del País Vasco (UPV/EHU), and Centro de Investigación Biomédica en Red en Enfermedades Neurodegenerativas (CIBERNED), 48940 Leioa, Spain

## Abstract

Neural regeneration resides in certain specific regions of adult CNS. Adult neurogenesis occurs throughout life, especially from the subgranular zone of hippocampus and the subventricular zone, and can be modulated in physiological and pathological conditions. Numerous techniques and animal models have been developed to demonstrate and observe neural regeneration but, in order to study the molecular and cellular mechanisms and to characterize multiple types of cell populations involved in the activation of neurogenesis and gliogenesis, investigators have to turn to* in vitro* models. Organotypic cultures best recapitulate the 3D organization of the CNS and can be explored taking advantage of many techniques. Here, we review the use of organotypic cultures as a reliable and well defined method to study the mechanisms of neurogenesis under normal and pathological conditions. As an example, we will focus on the possibilities these cultures offer to study the pathophysiology of diseases like Alzheimer disease, Parkinson's disease, and cerebral ischemia.

## 1. Introduction

The use of organotypic cultures in neuroscience research covers the experimental gap between the* in vitro* and* in vivo* models. It provides an opportunity to cultivate CNS tissue for weeks or months, giving open accessibility to complex cellular systems. Organotypic cultures are mainly prepared from P3–P10 animals (rats or mice) or, with some exception, from adult CNS tissues (e.g., [1]). Young postnatal animals already possess essential cytoarchitecture and are easy to handle with respect to the embryonic tissue, and nerve cells survive in the explants more than in adult slices. Nevertheless, the neurodegenerative diseases linked to ageing and adult brain present characteristics one cannot model with cultures from young animals. Particularly, young brain differs from the adult brain in terms of synaptic development and genetic and metabolic profiles. The first attempt to culture brain slices from adult rats and mouse failed because of their ability to reduce the thickness. Slices from young or perinatal animal can reduce their thickness from 350–400 mm to 100 mm after 1 or 2 weeks of incubation, while the mature adult slices nearly kept their thickness over a two-week cultivation period with consequent necrosis of the central cellular layers (see [1] for a review). Several technical clues were used to overcome and implement the technique to culture organotypic slices from adult. Progressive reduction of serum in the culture medium allowed increasing cell viability in 6–8-week-old mice cultures [2]. It is still unclear why complete withdrawal of serum resulted successfully in prolonging cell viability but one can speculate that negative effects of serum might be caused by excessive neurotrophic and energy resources [3].

The advantage of using organotypic cultures derives from their usefulness in experiments that require long-term survival, such as live recording [4, 5] or pharmacology (chronic drug application) as well as electrophysiology and optogenetics [6].

The first attempt to maintain CNS slice tissues in long-term cultures has been the “roller tube” technique [7]. This technique, finally characterized more in detail by Gähwiler and Knopfel [8, 9], was developed on the basis of experiences based on multitude of studies with explants culture [10].

In the roller tube cultures, the tissue is embedded in a plasma clot and attached on a glass coverslip. The coverslip with the embedded slice tissue is located in a tube that undergoes continuous slow rotation in a cell culture incubator. The oxygenation is maintained by continuous exchange of liquid-gas interface generated by the slow rotation. The technique was successively modified several times (e.g., [11, 12]), but the roller tube technique always yields very thin cultures (from an initial 400 *μ*m to about 50 *μ*m) with consequential preferential use for experiments that require optimal optical conditions (e.g., electron microscopy or electrophysiology).

**Figure 1:**
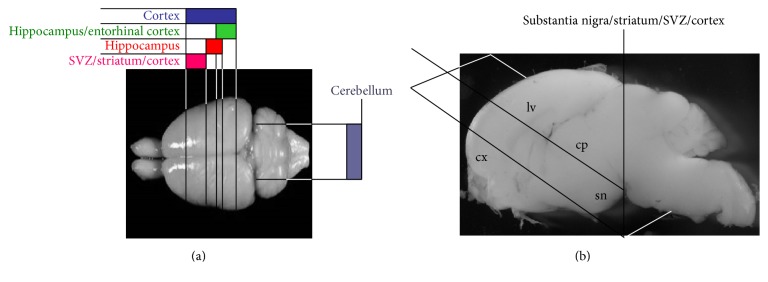
Different organotypic slices can be obtained from P2 to P7 postnatal forebrain or cerebellum by coronal sections of 350–400 *μ*m (a). In (b), the brain sectioning to obtain 45° transversal sections of s. nigra/striatum/SVZ/cortex (sn, substantia nigra; cp, caudate putamen; lv, lateral ventricle; cx, cortex) used to model PD is shown.

At the beginning of the 90s, Stoppini and colleagues [13] published a new method to cultivate organotypic slices. In this method, brain slices were placed on a semiporous membrane and cultivated at the air-liquid interface. The absence of clot facilitates the studies of synaptic reorganization and became a useful tool to study plasticity and sprouting already during the first days of culture. The real advantage of this technique is that cultures are easily prepared and offer great advantages when a 3D structure is desired (from an initial 400 *μ*m thickness, slices are cultivated up to 100–150 *μ*m). As described more in detail below, the air-liquid interface has become a key instrument to study adult neurogenesis. Organotypic slices can be obtained from different brain regions (as described in Figure 1), but for the study of adult neurogenesis in normal and pathological conditions, the hippocampal region containing the SGZ and the slices containing the SVZ are most preferred. Thus, for example, neurogenesis in Alzheimer disease or Parkinson's disease can be studied in hippocampus/entorhinal cortex or s. nigra/striatum/SVZ/cortex slices [14, 15]. Organotypic cultures match the tridimensional space where neural progenitors migrate to reach maturation* in vivo*. In the paper by Vergni and colleagues [16], we ideally represented the slice culture comprising subventricular zone, as the spatial extension to elaborate a mathematical model to describe neuroblasts activation and migration following oxygen and glucose deprivation.

## 2. Adult Neurogenesis in CNS

Neurogenesis in the normal adult brain occurs mainly in the dentate gyrus from the subgranular zone of hippocampus (SGZ) and in the olfactory bulb from the subventricular zone of lateral ventricle (SVZ). In both cases, the neurogenic niches, the SGZ and SVZ, host multipotent cells that give rise to neuroblasts or glioblasts throughout life. The stem cells in the neurogenic niches (type 1 in the SGZ or B cells in the SVZ) give rise to the more proliferative transit-amplifying progenitors (type 2 or C cells) that in turn give rise to neuroblast (type 3 or A cells) or glioblasts [17]. The study of adult neurogenesis in the SGZ is relevant especially for the study of repair mechanism of neurodegeneration related to loss of memory or neuropsychiatric disorders (for a review see [18]). New neuroblasts generated in the SGZ migrate to the granular zone of the dentate gyrus covering short distances, whereas neuroblasts generated in the SVZ move through the rostral migratory stream (RMS) to the olfactory bulb (OB) where finally they migrate radially and differentiate into new neurons.

Adult neural stem cells (NSC) are a specialized form of glia, deriving from the embryonic radial glia [19]. They possess a regional pattern within the SVZ which is related to their embryonic origin and reflect the ability to generate different types of OB interneurons (e.g., granule cells, TH+, or calbindin+ periglomerular cells; see [20]). The neurogenic moiety is guaranteed by different subpopulation of multipotent cells characterized by the expression of different markers in different temporal stages, as shown in Table 1. NSC can be activated, and neurogenesis can be induced, under different conditions. A predominant role* in vivo* is covered by the blood vessel and local vascular plexus that bring trophic factors or stress molecule signals. This was recently demonstrated by Katsimpardi et al. [21], who potentiated the neurogenesis of old animals after transfusion of blood from younger animals. The absence of local circulation in the organotypic cultures is fixed by using different trophic factors in the medium that stimulate intrinsic signals, mainly transcription factors, for example, sox2, olig2, or the bmp family [22, 23].

## 3. Techniques Used to Study Neurogenesis in OC

From a technical point of view, the organotypic cultures represent a versatile tool to study neurogenesis or cell regeneration.* In vivo* neurogenesis is a multistep process that involves proliferation, migration, and differentiation of neural stem cells as well as integration into preexisting network and functionality [24]. Each of the mentioned steps can be assayed in an organotypic slice. The method more used for studying the cell proliferation is the labelling with cell duplication markers. The most used ones are the nucleotide analogue 5-bromodeoxyuridine (BrdU) or 5-iododeoxyuridine (IdU) and the nuclear protein Ki67. BrdU and IdU incorporate into the duplicating DNA (during the S phase), whereas Ki67 protein is a nuclear protein expressed in all phases of cell duplication, all of which are subsequently visualized by immunofluorescence. In addition, the combination of BrdU and IdU can be used for time window experiments and cell characterization (for a review see [25]). Infection of fluorescent proteins with retroviruses is often used for time lapse experiments. Time set of different infections allowed the researcher to perform connectivity and lineage studies on newly generated cells directly* in vitro* [12, 26].

To study cell differentiation, BrdU/IdU immunofluorescence can be combined with antibodies for the different markers of differentiation shown in Table 1. Differentiation can be assayed also in organotypic cultures derived from transgenic animal expressing fluorescent reporter genes. This is the case of reporter mouse lines in which neural stem and progenitor cells express various fluorescent proteins (GFP, CFPnuc, H2B-GFP, DsRedTimer, and mCherry) under the control of the nestin promoter [27]. With these animals, one can follow all processes of differentiation and proliferation and evaluate the changes induced by various neurogenic and antineurogenic stimuli [28]. Using the fluorescent reporter genes is convenient also to verify the final integration of newly generated cells into a preexisting network by electrophysiology.

Organotypic cultures can be used especially to study the mechanisms of integration of new cells into preexisting circuitries. In the model proposed by Tønnesen et al. [29], they transplanted* in vitro* cultured GFP-TH neurospheres overexpressing embryonic Wnt5a into striatal organotypic slices. They observed neuronal differentiation (expression of neuronal markers and spontaneous firing of action potentials), synapse formation, and functional expression of dopamine D2 autoreceptors. In the same work, the authors could activate or inactivate optogenetically grafted cells demonstrating bidirectional synaptic interactions between grafted cells and host neurons and extensive synaptic connectivity within the graft.

## 4. Organotypic Cultures as a Model for CNS Neurodegeneration and Studies on Neurogenesis

### 4.1. Ischemia

Cerebral ischemia is generated by the loss of oxygen and nutrient in the brain.* In vivo* ischemia is generated in mice by occlusion of the middle cerebral artery that generates the damage of specific areas (cortex, striatum, and hippocampus), degeneration of neurons, and activation of microglia. In organotypic slices ischemic damage is modelled by oxygen and glucose deprivation (OGD). The neuronal damage generated is divided in a central core, where neurons die by necrosis, and a surrounding penumbra, where neurons die more slowly by apoptosis. This latter part sends death signals and starts a dual communication with the neurogenic niches. On one hand, the penumbra sends SOS signals to the SVZ which generates protective factors and activates neurogenesis [30]. On the other hand, death signals released from the focus of degeneration form a biochemical barrier impeding the neuroblasts migrating from SVZ to the damaged region. This was also modelled* in silico* by using cortex/SVZ/striatum cultures [16]. In this paper, we identified extracellular ATP and microglia activation as factors impeding neurogenesis and the interaction of SDF-1*α* with its receptor CXCR4 as a key signalling pathway driving neuroblasts migration.

The effect of neuroinflammation on neurogenesis after OGD has been largely studied in hippocampal organotypic cultures. In this culture, OGD generates a selective damage in the CA1 region and microglia recruitment to the damaged zone. Several papers demonstrated that anti-inflammatory treatment in organotypic slices facilitated the neurogenesis from the SGZ through the inhibition of both the p38 mitogen-activated protein kinase and metalloproteinases [31, 32]. Metalloproteinases are involved in the activation of several neuroinflammatory events. Also during OGD, this class of enzyme can sustain neuroinflammation and modulate neurogenesis in the dentate gyrus. In fact, the modulation of culture microenvironment after OGD in hippocampal organotypic cultures can promote the proliferation of glioblasts with predominant generation of oligodendrocyte progenitors [32].

### 4.2. Parkinson's Disease

Parkinson's disease (PD) is caused by the selective neurodegeneration of dopaminergic neurons. The main affected areas are the substantia nigra (s. nigra) and striatum. Nevertheless, dopaminergic degeneration affects also other areas like cerebral cortex, globus pallidus, and thalamus. The lack of dopamine and an unbalance of dopaminergic and glutamatergic signal cause the classical motor symptoms of bradykinesia, rigidity, resting tremors, and loss of postural reflexes. The system “s. nigra/striatum/cortex” has been reproduced* in vitro* with different organotypic cultures. The first attempt was made by Plenz and Kitai [33]; they cocultivated with the roller tube technique the three main areas involved in PD degeneration: cerebral cortex, striatum, and s. nigra. After a few days, the three regions were connected by new tyrosine hydroxylase positive fibers (TH^+^). Another coculture model to study dopaminergic degeneration was performed by combining the basal nucleus of Meynert, ventral mesencephalon, parietal cortex, and dorsal striatum of postnatal 7–9 rat pups [34]. Nevertheless, the presence of the SVZ in these organotypic slices is important beside the interest in adult neurogenesis. In fact, the SVZ is directly innervated by TH fibers suggesting that these can sustain the maintenance of the neurogenic niche. We described an organotypic model in which we maintained in one single slice the connection between s. nigra/striatum/cortex and SVZ [15]. In this culture, dopaminergic degeneration can be obtained either by mechanical lesion or by 6-OHDA injection. In both cases, the loss of dopamine stimulated the TH expression and proliferation of SVZ cells, suggesting the neurogenic niche can be activated following dopaminergic lesion. Moreover, while 6-OHDA generated more specific dopaminergic degeneration, the mechanical lesion can be used to study glutamatergic pathways or GABA-ergic degeneration which allows us to represent, respectively, early or advanced PD model (see [35] for a review).

### 4.3. Alzheimer's Disease

Alzheimer's disease (AD) is a chronic neurodegenerative disorder characterized by a progressive loss of memory and dementia. The causes and aetiology of AD are still unknown and the brain regions more affected are the hippocampus and neocortex. At molecular level, brain patients are characterized by amyloid plaques (dense and insoluble deposits of the beta-amyloid peptide-A*β*) and neurofibrillary tangles (intracellular aggregates of the hyperphosphorylated microtubule-associated protein tau). One of the first pieces of evidence of neurodegeneration in AD is the neuronal loss of cholinergic fibers of the entorhinal cortex connecting the CA1 layer of hippocampus [36]. AD is mimicked by different animal models from invertebrate [37] to rodents [38]. Except for the mouse SAMP8 [39], all transgenic models combine different mutations for amyloid precursor protein, tau, and presenilin 1.

The best organotypic model that represents cellular alteration observed in AD is the hippocampal slice. Alberdi and colleagues [14] used an organotypic slice in which they maintained the connection between entorhinal cortex and hippocampus. This model could be particularly useful to study cholinergic degeneration and activation of neurogenesis in the SGZ. Induction of neurodegeneration is obtained by treatment of slices with different A*β* peptides. As for several animal models, A*β* together with neuronal damage may play a role in the regulation of adult neurogenesis [4]. In particular, low concentration and the small A*β*_25–35_ peptide result in an increase of mossy fibers density and stimulation of endogenous SGZ neurogenesis. Nonetheless, neuroinflammation associated with A*β* toxicity can mediate the inhibition of SGZ neurogenesis through the release of various proinflammatory cytokines.

## 5. Other Organotypic Models for Neuroregeneration Studies

In addition to neurogenesis in the brain, organotypic cultures were employed also to study cell regeneration in spinal cord and PNS. Organotypic slices of mice adult spinal cord can be prepared with the liquid-air interface [40]. Spinal cord cells incorporate BrdU and express nestin, Oct3/4, and Dppa in the inner mass and have been used for modelling regeneration in spinal cord lesion [41]. Enteric neural stem cells (ENSC) have been isolated from adult intestine. They express markers like Ret, p75, and CD49b [42] and can differentiate primarily into glia [43] but also into neurons and myofibroblasts. If neurogenic potential of ENSC has been demonstrated* in vivo*, there is still discrepancy in results obtained* in vitro*.* Ex vivo* organotypic cultures from longitudinal muscle and myenteric plexus tissue demonstrated 5-ethynyl-2′-deoxyuridine incorporation in ENSC and potential proliferation dependent on the PTEN/PI3K/Akt pathway [44]. To investigate the neurogenesis in auditory system, Aburto and colleagues [45] used organotypic cultures of explanted chicken otic vesicles (OV). Neuroepithelial otic progenitors transit through states of cell proliferation, cell fate specification, cell cycle exit, migration, and differentiation showing characteristics of neural stem cells. In this case, autophagy covers a relevant role in maintaining clearance of apoptotic cells and facilitating neuronal differentiation of progenitor cells [45]. The inhibition of LC3B, a gene marker of autophagy, in organotypic cultures of OV provoked the misregulation of the cell cycle and impairing neurogenesis.

Other factors like GDNF can contribute to the final neuritogenesis of NSC-derived PNS neurons as demonstrated in organotypic slices of mice spiral ganglia [46]. Stimulation of GDNF-family receptor *α*-1 (GFR*α*1) activated two parallel pathways, PI3K/Akt and MEK/Erk especially during early postnatal days.

## 6. Concluding Remarks

Organotypic cultures of the neurogenic niches represent a valid alternative method to study neurogenesis in the CNS, which complements* in vivo* models and neurosphere cultures. Moreover, these cultures represent a good method to model brain damage in diseases like AD, PD, or stroke and to study how to implement neurogenesis as a potential mechanism of brain repair in these disorders. Organotypic slices have been used especially for toxicity studies; therefore, in neurogenesis research, they became valuable for testing drug effects on neurogenesis activation or improving cell fate specification.

## Figures and Tables

**Table 1:** SVZ neural stem cell characterization by cellular marker expression.

	GFAP	CD133	EGFR	Nestin	DCX	*β*III tubulin	NeuN	PDGFR	Proliferation
B cells									
qNSC	+++	++	−	−	−	−	−	−	−
aNSC	+++	++	++	+	−	−	−	+	+
C cells									
Transit amplifying	+	−	+	+++	−	−	−	++	+++
A cells									
Neuroblast	−	−	−	+	+++	+	−	−	++
Immature neurons	−	−	−	−	−	+++	+	−	+
Glioblast	+++	−	−	+	−	−	−	+++	+

Proliferation is expressed by BrdU incorporation ability (Codega et al., 2014) [17]. Quiescent neural stem cells, qNSC, and activated neural stem cells, aNSC.
